# Personalized CZA‐ATM dosing against an XDR *E. coli* in liver transplant patients; the application of the in vitro hollow fiber system

**DOI:** 10.1111/tid.14396

**Published:** 2024-11-04

**Authors:** Zahra Sadouki, Emmanuel Q. Wey, Satheesh Iype, David Nasralla, Jonathan Potts, Mike Spiro, Alan Williams, Timothy D. McHugh, Frank Kloprogge

**Affiliations:** ^1^ Institute for Global Health University College London London UK; ^2^ Centre of Clinical Microbiology University College London London UK; ^3^ Department of Infection Royal Free London NHS Trust London UK; ^4^ Department of HPB and Liver Transplant Surgery, Royal Free Hospital London UK; ^5^ Department of Hepatology Sheila Sherlock Liver Unit Royal Free London London UK; ^6^ Department of Surgical Biotechnology University College London London UK; ^7^ Department of Intensive Care Medicine Royal Free London London UK; ^8^ Department of Infection Sciences Health Services Laboratories London UK

**Keywords:** aztreonam, ceftazidime‐avibactam, *Escherichia coli*, hollow fiber infection model, personalized medicine, XDR

## Abstract

**Background:**

A patient with an extensively drug‐resistant (XDR) New Delhi metallo‐β‐lactamase (NDM) and oxacillinase (OXA‐48) producing *Escherichia coli (E. coli*) infection was awaiting orthotopic liver transplant. There is no standardized antibiotic prophylaxis regimen; however, in line with the Infectious Diseases Society of America guidance, an antibiotic prophylactic regimen of ceftazidime‐avibactam 2.5 g TDS with aztreonam 2 g three times a day (TDS) IV was proposed.

**Methods:**

The hollow fiber system (HFS) was applied to inform the individualized pharmacodynamic outcome likelihood prior to prophylaxis.

**Results:**

A 4‐log reduction in CFU/mL in the first 10 h of the regimen exposure was observed; however, the killing dynamics were slow and six 8‐hourly infusions were required to reduce bacterial cells to below the limit of quantification. Thus, the HFS supported the use of the regimen for infection clearance; however, it highlighted the need for several infusions. Standard local practice is to administer prophylaxis antibiotics at induction of orthotopic liver transplantation (OLT); however, the HFS provided data to rationalize earlier dosing. Therefore, the patient was dosed at 24 h prior to their OLT induction and subsequently discharged 8 days after surgery.

**Conclusion:**

The HFS provides a dynamic culture solution for informing individualized medicine by testing antibiotic combinations and exposures against the bacterial isolates cultured from the patient's infection.

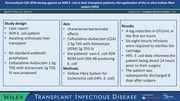

.

## Graphical abstract

The HFS‐*E. coli* informed the prophylactic antimicrobial treatment of an XDR‐*E. coli*‐infected patient awaiting orthotopic liver transplant. The patient was dosed 24 h prior to their surgery and subsequently discharged 8 days after surgery

## 1 INTRODUCTION

Antibiotic prophylaxis in the perioperative period is the standard of care and routinely prescribed during surgical procedures including solid organ transplantation (SOT). Although evidence suggests an overall benefit in reducing post‐operative infections, the standard of care regimen and duration vary between transplant centers.^1^, ^2^, ^3^ Here, we present the application of the hollow fiber system for *Escherichia coli* (HFS‐*E. coli*) to inform perioperative personalized antimicrobial dosing in a complex clinical setting where a patient with a history of extensively drug‐resistant (XDR) New Delhi metallo‐β‐lactamase (NDM) and oxacilinase (OXA‐48) producing *E. coli* infection, inadequate source control (billomas), and prosthetic material colonization (biliary stents and drain) was awaiting orthotopic liver transplant. In these circumstances, a standardized antibiotic prophylaxis regimen may not provide adequate suppressive prophylactic cover and antimicrobial combination therapy will require case‐by‐case–based selection, accounting for individual risk factors and local patterns of resistance, given the rising incidence of multidrug‐resistant infections in solid organ transplant donors and recipients. Systematic reviews and meta‐regression analysis have indicated that there is a statistically significant risk associated with gastrointestinal tract colonization of carbapenem‐resistant enterobacterales (CRE) and subsequent infection with CRE.^4^

Informing personalized dosing requires in vitro testing beyond minimum inhibitory concentration (MIC) reporting, in which XDR and pan‐drug resistant (PDR) infections only serves to highlight the limited options of antimicrobials.^5^ In these contexts, HFS‐*E. coli* provides an in vitro tool that can inform perioperative dosing through mimicking in vivo like pharmacokinetic profiles in order to study bacterial killing. Semi‐permeable hollow fibers, packed in a cylinder, retain the bacteria in the extra capillary space (ECS) while allowing for a free flow of nutrients and drugs.^6^ Unlike animal experiments, that are often used in drug development to characterize bacillary clearance, HFS‐*E. coli* offers longitudinal sampling without the cost or ethical approval required for animal studies. Therefore, the bacteria isolated from the patient can be challenged against the expected in vivo antimicrobial exposures in the patient to investigate bacterial killing ahead of treatment.^6^

For this particular case, previous infection had been controlled but amid fear of post‐operative regrowth as a consequence of possible seeding and contamination of the operative field at the time of liver explantation and removal of colonized biliary stents and drain, targeted prophylaxis was needed. The aim of this study was to characterize bactericidal effects with ceftazidime‐avibactam (CZA) 2.5 g TDS with aztreonam (ATM) 2 g TDS IV and the patients’ own *E. coli* XDR, NDM, and OXA‐48 producing *E. coli* strain prior to orthotopic liver transplantation (OLT) to inform antimicrobial prophylaxis.

## 2 MATERIALS/PATIENTS AND METHODS

### 2.1 Information on clinical findings and diagnostic assessment

An inpatient 45‐ to 55‐year‐old male at University College London Hospitals NHS Foundation Trust (UCLH), also under the care of hepatology and hepatobiliary surgery teams at Royal Free London NHS Foundation Trust (RFL), previously diagnosed with IgG‐related sclerosing cholangitis, presented with recurrent biliary sepsis over a 3‐month period (Table 1). Source control of the infections was not achieved by empirical antibiotic treatment due to the presence of three metallic stents in his biliary tree and an internalized percutaneous biliary drain. Blood culture samples and testing of the perihepatic collection fluid confirmed several multi‐drug resistant (MDR), NDM, and OXA‐48 positive Gram‐negative bacterial strains (Table S1) with a combined resistance across nine major antibiotic classes. Further complicating the patient's clinical management was the damage to the liver and loss of synthetic function at the time the patient was placed on the NHS waiting list for OLT. The Infectious Diseases Society of America (IDSA) guidance on the treatment of antimicrobial‐resistant Gram‐negative infections particularly metallo‐β‐lactamase (MBL) prodiucing carbapenem resistant enterobacterales CRE (e.g., NDM, imipenemase (IMP), and Verona integron‐encoded metallo‐ß‐lactamase (VIM)) suggests intravenous three times daily dosing of 2.5 g CZA in addition to 2 g ATM.^7^ In addition, several clinical infectious disease publications report efficacy of ceftazidime (CZA) plus aztreonam (ATM) in patients with blood stream infections (BSIs).^8^, ^9^ Further antibiotic susceptibility testing (AST) revealed CZA‐ATM synergy where ATM activity was restored when in combination with avibactam (AVI). Therefore, the HFS‐*E. coli* was employed with the most difficult to treat XDR strain, namely, XDR *E. coli* (Table 2), as a proof of concept to simulate the in vivo dynamic concentrations of the proposed treatment of a three‐way antibiotic dosing regimen to be administered to a clinically complex patient. These included ATM 2 g every 8 h given as a 1‐h infusion and CZA 2.5 g 8 hourly over 2 h for a total duration of 5 days.

**TABLE 1:** Patient clinical details and timeline.

Clinical scores	Value (units)	Context
SOFA score	0	At transplantation
MELD score	10	At transplantation
APACHE II score	2	At transplantation

Laboratory indices		Context
Total bilirubin	9 (μmol/L)	At transplantation
ALT	120 (U/L)	At transplantation
AST	93 (U/L)	At transplantation
Creatinine	99 (μmol/L)	At transplantation
GFR	75 (mL/min)	At transplantation

Infective episode (organism)/colonization	Treatment duration	Context
*E coli* XDR NDM and OXA‐48 biliary sepsis with bacteraemia	6 weeks	17 months pre‐transplant isolated from blood stream and bile
Repeated colonization with E Coli NDM OXA‐48	n/a IPC measures in place	Colonization from rectal, drain, and surgical sterile and non‐sterile sites. 22 months pre‐transplantation until 1 week post transplantation
*E coli* XDR NDM and OXA‐48 surgical site infection (SSI) following instrumentation of biliary tree and stent replacement	10 days	18 months pre‐transplant
*E coli* and *P mirabilis* biliary sepsis with bacteraemia	2 weeks	36 months pre‐transplant

**TABLE 2:** Minimum inhibitory concentrations (MICs) observed for extensively drug‐resistant (XDR) *E. coli* isolate and NCTC 12241 type *E. coli* versus EUCAST clinical breakpoints and EUCAST quality control (QC) ranges.

Antibiotic/s	*E. coli* XDR isolate modal (HFIM_1_BSI) MIC (μg/mL)	EUCAST clinical breakpoint μg/mL	*E. coli* ATCC 25922 modal MIC μg/mL	EUCAST ATCC 25922 QC ranges μg/mL
CAZ	>80	R (>4)	0.16	0.06–0.5
AVI	>14	–	>14	n/a
ATM	>120	R (>4)	0.125	0.06–0.25
CZA^a^	>32/4	R (>8/4)	0.25/4	0.06–0.5
ATM‐AVI*	2/4	–	0.125/4	n/a
CZA‐ATM	2.5/0.44/3.8	‐	<0.31/0.05/0.47	n/a

*Note*: ATM‐AVI refers to aztreonam MIC (μg/mL)/avibactam MIC (μg/mL). CZA‐ATM refers to ceftazidime MIC (μg/mL)/avibactam MIC (μg/mL)/aztreonam MIC (μg/mL). MICs, obtained following CLSI guidelines, achieved for antibiotic/s tested are presented in columns one (XDR *E. coli*) and three (NCTC *E. coli*). MICs are shaded in red to represent resistant clinical breakpoints category ranges for enterobacterales. All MICs for the reference laboratory *E. coli* strain (ATCC 25922) fell within EUCAST quality control ranges.

Abbreviations: CAZ, ceftazidime, ATM, aztreonam; AVI, avibactam.

^a^ Avibactam concentration fixed at 4 μg/mL.

### 2.2 Informed consent

Health Research Authority Decision Panel advisors did not consider the work research but a deviation from standard of care that was purely for clinical needs. Review by an NHS Research Ethics Committee was therefore not required. The patient was consented for orthotopic liver transplantation and normothermic perfusion as it is a standard for all patients and includes consent for outcomes of research publications, and the use of peri‐transplant tissue samples for research. In this case, no patient or deceased donor tissue was used for in vitro testing.

### 2.3 Laboratory consumables and experimental setup

The laboratory control strain (ATCC 25922) and the clinical isolate (XDR *E. coli*) were prepared identically. Each was cultured at 37°C in ambient air on nutrient agar and cryopreserved in Microbank beads at ‐80C. MICs were determined following CLSI guidelines for microbroth dilution.^10^ A mid‐log phase inoculum for each isolate was prepared for the HFS‐*E. coli* by refreshing a fresh overnight culture incubated in a shaking incubator at 200 rpm and 37C in cation adjusted Mueller Hinton broth (CAMHB; Sigma). A 1:50 dilution from this culture was performed to inoculate the HFS‐*E. coli* ECS, achieving a final bacterial load of 10^5^ CFU/mL in the ECS.

The R‐Shiny web application promoted by the International Society of Anti‐Infective Pharmacology (ISAP) and ESCMID pharmacokinetic/pharmacodynamic modeling (PK/PD) of anti‐infectives study group (EPASG) was used to define experimental setup parameters.^11^ The following *C*_max_ concentrations were mimicked: CAZ 80 μg/L, AVI 14 μg/L, and ATM 120 μg/L. A *T*_½_ of 2.48 h was simulated in the HFS‐*E. coli* (Table S2 and Figure S1). Antibiotic powders were dissolved in solvents recommended by the manufacturers (CAZ; Sigma, AVI; MCE, and ATM; TOKU‐E).

CAMHB (Sigma) was broth media that was used in the HFS‐*E. coli* system. The hollow fiber cartridge was sourced from FibreCell systems (High flux PS, C2011). The FibreCell duet pump was set at 100 mL/min to allow the cartridge and central reservoir to reach equilibrium. A peristaltic pump (Minipuls evolution, Gilson) was set at 0.699 mL/min to mimic *T*_½_ of 2.48 h. The syringe driver pumps (AL‐1000, WPI) were programmed to automatically dose 2 mL infusions every 8 h over 2 h and 1 h, respectively, for CZA and ATM; administration was concurrent and direct into the central reservoir. Dosing timepoints were at 0, 8, 16, 24, 32, 40, 48, 56, 64, 72, 80, 88, 96, 104, and 112.

The cartridge ECS was sampled on Mueller Hinton agar (MHA) Avantor (VWR) to quantify CFUs as well as on Colombia blood agar (VWR) to confirm the absence of contamination. The experiments with the laboratory control strain (ATCC 25922) and the clinical isolate (XDR *E. coli*) were both performed in biological duplicate and all bacterial CFU/mL measurements were performed in technical duplicates at timepoints 0, 2, 4, 6, 8, and 10 to capture the initial killing dynamics again, every 24 h for 5 days to capture sustained killing or regrowth.

## 3 RESULTS

MICs demonstrated synergy between ATM‐AVI against the XDR *E. coli* isolated from the patient's blood stream (Table 2). Although Clinical & Laboratory Standards Institute (CLSI) and European Committee on Antimicrobial Susceptibility Testing (EUCAST) are yet to establish breakpoints for the ATM‐AVI combination, this MIC and synergy data seen fell within range of previous in vitro studies reporting MICs between ≤0.03/4 μg/mL and 8/4 μg/mL, most typically ≤2/4 μg/mL for MBL‐producing enterobacterales.^12^, ^13^

**FIGURE 1:**
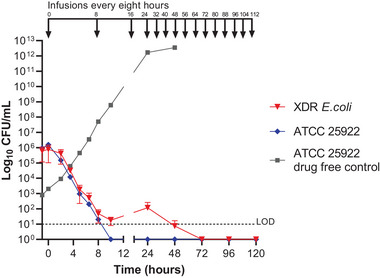
Extensively drug‐resistant (XDR) *E*. *coli* against Ceftazidime/Avibactam/Aztreonam combination regimen in HFS‐*E. coli*. Total bacterial population counts in CFU/mL over time. Data points for XDR *E. coli* (red) represent the geometric mean ± SD of biological duplicates. Antibiotic infusions were dosed every 8 h (shown in black at the top of the graph). The lower limit of detection (LOD) for quantifying the bacteria was 10 CFU/mL and shown as black dashed line.

The XDR *E. coli* challenged with CZA‐ATM combination therapy infusions produced a steady rate of killing for the first 10 h with a 4 log reduction in bacterial CFU/mL; however, six 8‐hourly infusions were required to reduce bacterial cells to below the limit of quantification (Figure 1). Therefore, multiple infusions were required to achieve bacterial source control. The HFS‐*E. coli* data demonstrated the proposed combination therapy was only effective at clearing the XDR *E. coli* after 2 days of administration of 8‐hourly infusions. In effect this suggests longer extended infusions or earlier administration of prophylaxis would be necessary for this patient; toward the end of the infusion, the simulated concentrations of both ATM and CAZ drop below their respective MICs.

In general, clamping of the inferior vena cava occurs at 2 to 3 h after induction, followed by a anhepatic period from 3 to 4 h post induction of the OLT, during which the mobilization and explantation of the native liver takes place. It would be at this point when mobilization and removal of the primary prosthetic sources of XDR biofilm would take place. Surgical implantation of the new deceased donor graft would, therefore, theoretically coincide with the MIC nadir and potential bacteraemia resulting from explantation of the native liver, and if the first dose was given at induction it would also coincide with a CFU/mL load above the limit of detection (LOD). This informed the patients antibiotic prophylaxis, and ceftazidime avibactam 2.5 g Iv and Aztreonam 2 g IV were first dosed at 12 h prior to the anhepatic phase of their OLT induction, thus different from the standard local practice of administering prophylaxis antibiotics at the induction of OLT. In addition the duration of prophylaxis was extended from the standard 24 hours to 48 hours. The patient received a donnation after circulatory death (DCD) graft with side‐to‐side piggyback anastomosis, portal vein end‐to‐end anastomosis, and Roux‐en‐Y hepaticojejunostomy with removal of a previously internalized percutaneous biliary drain.

The patient was discharged 8 days after surgery and had no reoccurrence of XDR‐*E. coli* infection reported in the year after follow up. Incidentally, the patient has also remained CRE negative on screening post OLT.

## 4 CONCLUSIONS

Several attempts have been made to develop in vitro laboratory susceptibility testing methods for the combination of CZA with ATM against XDR Gram‐negative bacteria.^14^, ^15^, ^16^ However, these methods test static antibiotic concentrations. Here, we challenged a patient XDR BSI isolate against dynamic in vivo simulated concentrations of the CZA‐ATM antibiotic combination. We used the HFS‐*E. coli* in real time to generate data that demonstrated the CZA‐ATM antibiotic regimen was effective at clearing the bacterial cells below the limit of detection in the hollow fiber ECS; however, multiple infusions were required. Moreover, an earlier start than standard practice was needed, that is, 8 h preoperative, to ensure adequate bacterial killing before the induction of the surgical procedure. The Royal Free London Trust standard of care is to administer prophylactic antibiotics for OLT at induction.^3^ However, the slow killing dynamics observed in the HFS‐*E. coli* provided in vitro evidence to support prophylactic administration prior to OLT as well as an extended prophylactic duration of 48 h. Therefore, the patient awaiting a liver transplant was dosed 12 h earlier than standard local practice. The patient was subsequently discharged 8 days after OLT. The unpredictability related to the timing and lead times associated with deceased donor grafts complicates the practicality of commencing antibiotic prophylaxis in this patient cohort far in advance of 12 h. This is compounded by the requirement to administer both ceftazidime‐avibactam and aztreonam concomitantly.

The increased application of alternative synergistic combinations of antibiotic agents necessitates improved methodologies and approaches to in vitro evaluation of combination regimens. We demonstrate that the extended prophylaxis periods are superior for a chronically infected patient where source control has not been achieved with conventional antibiotic treatment and alternative combination regimens are used. The need of standardized methods to support personalized medicine will be increased as the antibiotic resistance era heightens. The HFS‐*E. coli* provides a solution for testing different antibiotic regimens against the exact bacterial isolate cultured from the patient. Specific PK concentration profiles of the proposed antibiotics can be mimicked. The increased prescribing of alternative synergistic combinations necessitates improved in vitro methodology for evaluating personalized dosing. The HFS‐*E. coli* provides a dynamic culture solution for testing personalized antibiotic regimens and exposures against the bacterial isolates cultured from the patient. This could inform targeting therapy, which could preserve antimicrobials and uphold antimicrobial stewardship.

## CONFLICT OF INTEREST STATEMENT

The authors declare no conflicts of interest.

## Supporting information



Supporting information

Visual Abstract

## Data Availability

All data are available upon reasonable request.

## References

[1] Anesi JA , Blumberg EA , Abbo LM . Perioperative antibiotic prophylaxis to prevent surgical site infections in solid organ transplantation. Transplantation. 2018;102:21‐34. 28614192 10.1097/TP.0000000000001848

[2] Abbo LM , Grossi PA . Surgical site infections: guidelines from the American Society of Transplantation Infectious Diseases Community of Practice. Clin Transplant. 2019;33(9) e13589. 31077619 10.1111/ctr.13589

[3] Campos‐Varela I , Blumberg EA , Giorgio P , et al. What is the optimal antimicrobial prophylaxis to prevent postoperative infectious complications after liver transplantation? A systematic review of the literature and expert panel recommendations. Clin Transplant. 2022;36:e14631. 35257411 10.1111/ctr.14631

[4] Willems RPJ , Van Dijk K , Vehreschild MJGT , et al. Incidence of infection with multidrug‐resistant Gram‐negative bacteria and vancomycin‐resistant enterococci in carriers: a systematic review and meta‐regression analysis. Lancet Infect Dis. 2023;23:719‐731. 36731484 10.1016/S1473-3099(22)00811-8

[5] Gajic I , Kabic J , Kekic D , et al. Antimicrobial susceptibility testing: a comprehensive review of currently used methods. Antibiotics(Basel). 2022;11(4)427. 35453179 10.3390/antibiotics11040427 PMC9024665

[6] Sadouki Z , McHugh TD , Aarnoutse R , et al. Application of the hollow fibre infection model (HFIM) in antimicrobial development: a systematic review and recommendations of reporting. J Antimicrob Chemother. 2021;76(9):2252‐2259 doi:10.1093/jac/dkab160 34179966 PMC8361333

[7] Tamma PD , Heil EL , Justo, JA , et al. IDSA guidance on the treatment of antimicrobial‐resistant Gram‐negative infections. Clin Infect Dis. 2024; ciae403. 10.1093/cid/ciae403 39108079

[8] Falcone M , Daikos GL , Tiseo G , et al. Efficacy of ceftazidime‐avibactam plus aztreonam in patients with bloodstream infections caused by metallo‐β‐lactamase–producing enterobacterales. Clin Infect Dis. 2021;72:1871‐1878. 32427286 10.1093/cid/ciaa586

[9] Yasmin M , Fouts DE , Jacobs MR , et al. Monitoring ceftazidime‐avibactam and aztreonam concentrations in the treatment of a bloodstream infection caused by a multidrug‐resistant enterobacter sp. carrying both klebsiella pneumoniae carbapenemase–4 and New Delhi metallo‐β‐lactamase–1. Clin Infect Dis. 2020;71:1095. 31802119 10.1093/cid/ciz1155 PMC7428388

[10] CLSI . M100: Performance Standards for Antimicrobial Susceptibility Testing. 33rd ed. CLSI; 2023.

[11] Aranzana‐Climent V , Chauzy A , Grégoire N . HF‐App: A R‐Shiny application to streamline hollow‐fibre experiments. R application version 1.0.0. 2021. https://varacli.shinyapps.io/hollow_fiber_app/

[12] Lutgring JD , Balbuena R , Reese N , et al. Antibiotic susceptibility of NDM‐producing enterobacterales collected in the United States in 2017 and 2018. Antimicrob Agents Chemother. 2020;64(9):e00499‐20. 10.1128/aac.00499-20 32540972 PMC7449154

[13] Chauzy A , Buyck J , De Jonge BLM , Marchand S , Grégoire N , Couet W . Pharmacodynamic modelling of β‐lactam/β‐lactamase inhibitor checkerboard data: illustration with aztreonam‐avibactam. Clin Microbiol and Infect. 2019;25:515‐516. 10.1016/j.cmi.2018.11.025 30543853

[14] Rawson TM , Brzeska‐Trafny I , Maxfield R , et al. A practical laboratory method to determine ceftazidime‐avibactam‐aztreonam synergy in patients with New Delhi metallo‐beta‐lactamase (NDM)–producing Enterobacterales infection. J Glob Antimicrob Resist. 2022;29:558‐562. 35131508 10.1016/j.jgar.2022.01.025

[15] Sreenivasan P , Sharma B , Kaur S , et al. In‐vitro susceptibility testing methods for the combination of ceftazidime‐avibactam with aztreonam in metallobeta‐lactamase producing organisms: role of combination drugs in antibiotic resistance era. J Antibiot. 2022;75:8:454‐462. 10.1038/s41429-022-00537-3 PMC9204069 35715617

[16] Khan A , Erickson SG , Pettaway C , Arias CA , Miller WR , Bhatti MM . Evaluation of susceptibility testing methods for aztreonam and ceftazidime‐avibactam combination therapy on extensively drug‐resistant Gram‐negative organisms. Antimicrob Agents Chemother. 2021;65. 10.1128/aac.00846-21 PMC8522751 34424044

