# Supplementary material for: Personalized CZA‐ATM dosing against an XDR E. coli in liver transplant patients; the application of the in vitro hollow fiber system
Source: Transpl Infect Dis. 2024 Nov 4;27(1):e14396. doi: 10.1111/tid.14396 (PMC11827718; doi:10.1111/tid.14396)
Supplement: Supplementary file 1 — Supporting information [file TID-27-e14396-s002.docx]

Supplementary data: Personalized CZA-ATM dosing against an XDR *E. coli* in liver transplant patients; the application of the *in vitro* hollow fibre infection model (HFIM)

Zahra Sadouki ^1, 2^,^ǂ^ Emmanuel Q. Wey ^2, 3 ǂ^, Sateesh Iype^4^, David Nasralla^4^, Jonathan Potts ^5^, Mike Spiro ^7,8^ Alan Williams^6^ Timothy D. McHugh ^2^, Frank Kloprogge^1^

1 Institute for Global Health, University College London, UK

2 Centre of Clinical Microbiology, University College London, UK

3 Department of Infection, Royal Free London NHS Trust, UK

4 Department of HPB and Liver Transplant Surgery Royal Free Hospital

5 Department of Hepatology, Sheila Sherlock Liver Unit, Royal Free London

6 Department of Infection Sciences, Health Services Laboratories, London, UK,

7 Department of Surgical biotechnology, UCL

8 Department of Intensive care medicine Royal Free London

^ǂ^ Both authors contributed equally

**Corresponding author:** Dr Emmanuel Wey, Department of Infection, Consultant and Honorary Associate Professor in Infection, Royal Free London NHS Trust, Centre for Clinical Microbiology, Division of Infection & Immunity, University College London

[emmanuel.wey@nhs.net](mailto:emmanuel.wey@nhs.net)

**Table of Contents**

[Mean inhibitory concentrations (MICs) observed 3](#_Toc168991181)

[MICs observed against all cultures isolated from the liver transplant patient 3](#_Toc168991182)

[MICs observed against BSI isolate 5](#_Toc168991183)

[HFIM methodology 6](#_Toc168991184)

[CZA-ATM concentrations simulated in the HFIM 7](#_Toc168991185)

# Mean inhibitory concentrations (MICs) observed

## MICs observed against all cultures isolated from the liver transplant patient

| Table S1. MICs μg/mL observed against cultures isolated from the liver transplant patient. MIC’s were obtained using standard incoculla nad automated AST platforms (BD-Phoenix) using EUCAST breakpoints. Additional testing modalities include EUCAST e -test methodologies and the CARBA-5 lateral flow assay. Sequencing was performed at UKHSA Colindale AMRHAI. Red represents isolates in the resistant category and green represents isolates in sensitive category as per EUCAST clinical breakpoints for Enterobacterales. Isolate ‘XDR *E.* coli’ was used in the HFIM experiments presented in Figure 1. | | | | | |
| --- | --- | --- | --- | --- | --- |
| **Antibiotic** | **XDR *E. coli*** | **HFIM_2_Drain** | **HFIM_3_Drain** | **HFIM_4_Drain** | **HFIM_5_Abscess** |
|  | ***E. coli*** | ***E. coli*** | ***E. coli*** | ***M. morganii*** | ***E. coli*** |
| Ampicillin | >8 | >8 | >8 | >8 | >8 |
| Amikacin | <=4 | <=4 | 8 | <=4 | 8 |
| Aztreonam | >16 | 8 | >16 | 4 | >16 |
| Co-Amoxiclav | >32/2 | >32/2 | >32/2 | >32/2 | >32/2 |
| Ceftazidime | >16 | >16 | >16 | 2 | >16 |
| Cefixime | >2 | >2 | >2 | >2 | >2 |
| Ciprofloxacin | >1 | >1 | >1 | >1 | >1 |
| Colistin | <=1 | <=1 | <=1 | >4 | <=1 |
| Cephalexin | >16 | >16 | >16 | >16 | >16 |
| Ceftriaxone | >4 | >4 | >4 | >4 | >4 |
| Cefuroxime | >8 | >8 | >8 | >8 | >8 |
| Ertapenem | >1 | <=0.25 | >1 | <=0.25 | <=0.25 |
| Cefepime | >16 | <=1 | >16 | 2 | >16 |
| Nitrofurantoin | <=16 | <=16 | <=16 | 128 | <=16 |
| Fosfomycin | <=16 | <=16 | <=16 | 64 | 32 |
| Gentamicin | >4 | 2 | >4 | >4 | - |
| Imipenem | >8 | <=0.25 | >8 | 8 | 2 |
| Levofloxacin | >2 | >2 | >2 | >2 | 1 |
| Mecillinam | >8 | <=2 | >8 | - | >2 |
| Meropenem | >8 | <=0.125 | >8 | 0.25 | 0.25 |
| Nalidixic acid | >16 | >16 | >16 | >16 | >16 |
| Tobramycin | >4 | 2 | >4 | >4 | >4 |
| Piperacillin | >8 | >64 | >8 | >8 | >8 |
| Norfloxacin | >64 | >2 | >64 | - | >64 |
| Co-trimoxazole | >4/76 | >4/76 | >4/76 | >4/76 | <=1/19 |
| Temocillin | >32 | 16 | >32 | - | >32 |
| Tigecycline | 1 | <=0.5 | 1 | 2 | 1 |
| Ticarcillin/clavulanate | >64/2 | >64/2 | >64/2 | >64/2 | >64/2 |
| Trimethoprim | >4 | >4 | >4 | >4 | >4 |
| Piperacillin/tazobactam | >64/4 | <=4/4 | >64/4 | <=4/4 | >64/4 |

# HFIM methodology

| **Table S2. HFIM experimental set up including technical and microbiological specifications** | | |
| --- | --- | --- |
| Section | HFIM feature | Further explanation |
| Descriptive specifications | Primary aim | Mimic antibiotic combination therapy proposed for patient |
|  | Microbial species | XDR *E. coli* inoculated into extra-capillary space (ECS) |
|  | Antimicrobial/s | Ceftazidime + avibactam + aztreonam |
|  | Duration | 120 hours (5 days) |
| Technical specifications | Mimicked dose | 2.5g CZA over 2hr + 2g ATM over 1hr |
|  | System volume | 150 mL (central 80 mL + cartridge 70 mL) |
|  | C_max_ | Ceftazidime 80 µg/L (∴ administered 7.8 µg/L)  Avibactam 14 µg/L (∴ administered 1.4 µg/L)  Aztreonam 120 µg/L (∴ administered 1.4 µg/L) |
|  | T_1/2_ | 2.48 hours |
|  | Τ | 8 hours dosing interval |
|  | Drug administration | Syringe infusion (automated driver pump) |
|  | Cartridge source | FibreCell Systems Cartridge C2011 |
|  | Fibre type | High flux polysulfone |
|  | Pump model | FiberCell Systems® Inc duet pump at ~100 mL/min  Gilson pump set at 0.699 mL/min (5.92 rpm) |
| Microbiological specifications | Media | Mueller Hinton Broth 2, Cation-Adjusted (Sigma; 90922) |
|  | Control | Drug free experiment conducted in duplicate |
|  | Contamination | Sampling of ECS and reservoirs on CBA (VWR; 89407-240) |
|  | Inoculum | Initial OD600 for approximation then CFU/mL quantified |
|  | CFUs | CFUs quantified on MHA (VWR; 100611ZA) |
|  | Biological repeat | Duplicates |
|  | Technical repeat | Duplicates |

# CZA-ATM concentrations simulated in the HFIM

| 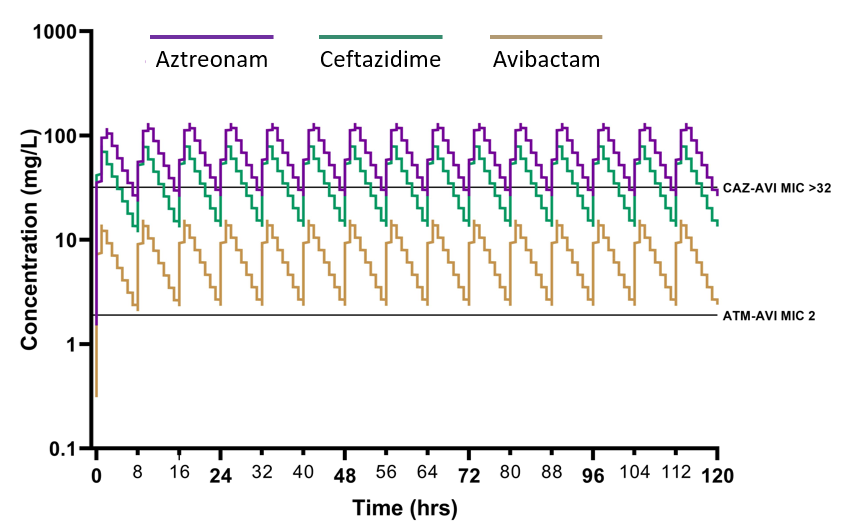 |
| --- |
| **Figure S1. HFIM simulated concentrations of CZA-ATM**  Dynamic concentrations of aztreonam (ATM), ceftazidime (CAZ) and avibactam (AVI) simulated in the HFIM experiments using *in vivo* PK data. Solid lines on the y axis represent the MIC for each antibiotic/combination tested in static microbroth dilution. |
