# Supplementary material for: Personalized CZA‐ATM dosing against an XDR E. coli in liver transplant patients; the application of the in vitro hollow fiber system
Source: Transpl Infect Dis. 2024 Nov 4;27(1):e14396. doi: 10.1111/tid.14396 (PMC11827718; doi:10.1111/tid.14396)
Supplement: Supplementary file 2 — Visual Abstract [file TID-27-e14396-s001.pptx]

## Slide 1
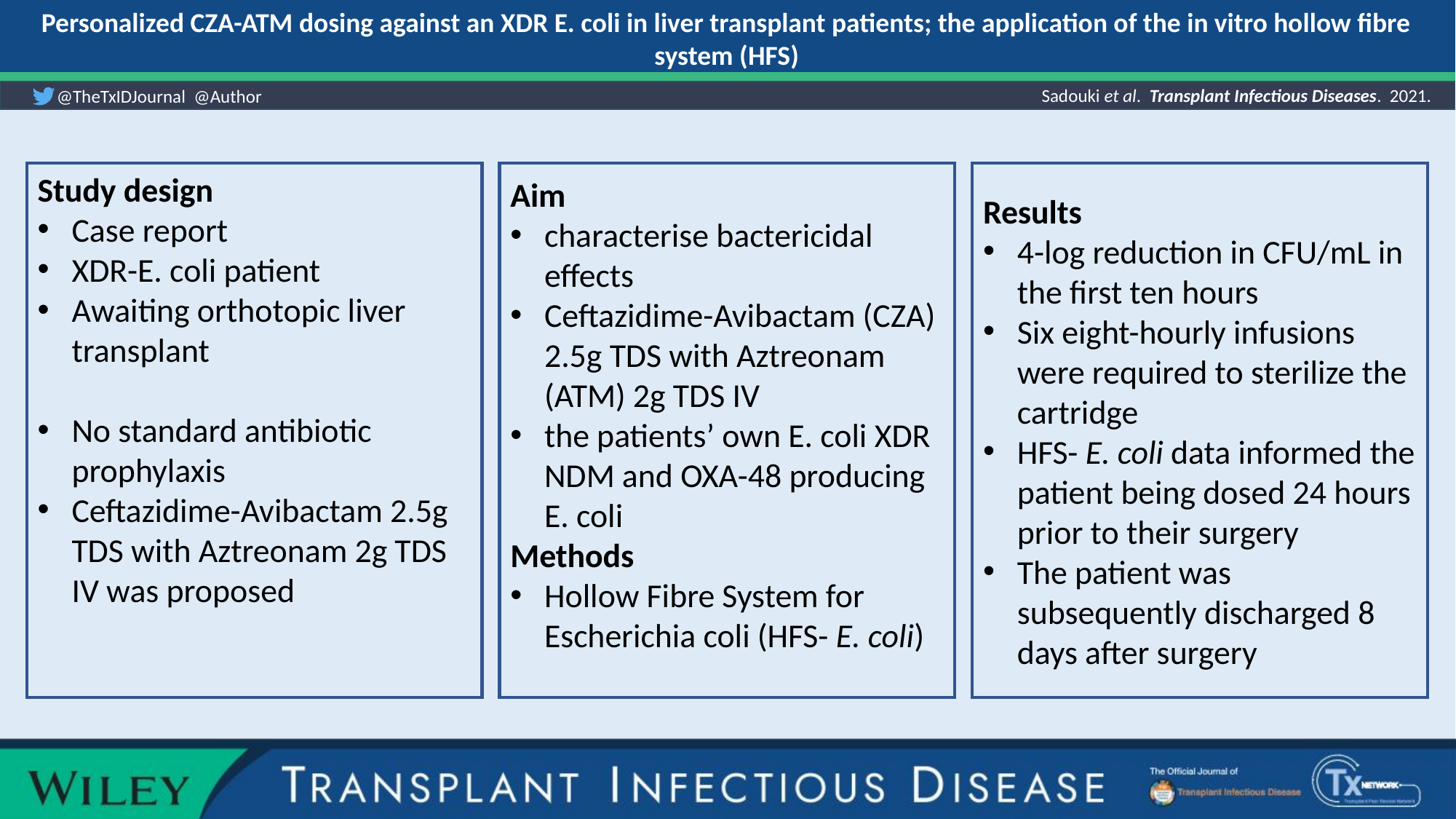

Personalized CZA-ATM dosing against an XDR E. coli in liver transplant patients; the application of the in vitro hollow fibre system (HFS)
Sadouki et al. Transplant Infectious Diseases. 2021.
 @TheTxIDJournal @Author
Study design
Case report
XDR-E. coli patient
Awaiting orthotopic liver transplant
No standard antibiotic prophylaxis
Ceftazidime-‎Avibactam 2.5g TDS with Aztreonam 2g TDS IV was proposed
Aim
characterise bactericidal effects
Ceftazidime-‎Avibactam (CZA) 2.5g TDS with Aztreonam (ATM) 2g TDS IV
the patients’ own E. coli XDR NDM and OXA-48 producing E. ‎coli
Methods
Hollow Fibre System for Escherichia coli (HFS- E. coli)
Results
4-log reduction in CFU/mL in the first ten hours
Six eight-hourly infusions were required to sterilize the cartridge
HFS- E. coli data informed the patient being dosed 24 hours prior to their surgery
The patient was subsequently discharged 8 days after surgery
